# High-Precision Fiber Noise Detection and Comparison over a 260 km Field Fiber Link

**DOI:** 10.3390/s24113483

**Published:** 2024-05-28

**Authors:** Qi Zang, Xiang Zhang, Dan Wang, Qian Zhou, Le Fan, Yucan Zhang, Ru Yuan, Jing Gao, Dongdong Jiao, Guanjun Xu, Tao Liu, Ruifang Dong, Shougang Zhang

**Affiliations:** 1 National Time Service Center, Chinese Academy of Sciences, 3 Shuyuandong Road, Xi’an 710600, China; zangqi@ntsc.ac.cn (Q.Z.); zhangxiang@ntsc.ac.cn (X.Z.); wangdan@ntsc.ac.cn (D.W.); zhouqian@ntsc.ac.cn (Q.Z.); fanle@ntsc.ac.cn (L.F.); zhangyucan@ntsc.ac.cn (Y.Z.); yuanru@ntsc.ac.cn (R.Y.); gaojing@ntsc.ac.cn (J.G.); jandan19@sina.com (D.J.); xuguanjun@ntsc.ac.cn (G.X.); taoliu@ntsc.ac.cn (T.L.); szhang@ntsc.ac.cn (S.Z.); 2 Key Laboratory of Time Reference and Applications, Chinese Academy of Sciences, 3 Shuyuandong Road, Xi’an 710600, China; 3 University of Chinese Academy of Sciences, 19 Yuquan Road, Beijing 100049, China

**Keywords:** frequency comparison, fiber noise sensing, optical frequency transfer, field fiber link

## Abstract

In this paper, we present a high-precision optical frequency noise detection and comparison technique using a two-way transfer method over a 260 km field fiber link. This method allows for the comparison of optical frequencies between remote optical references without the need for data transfer through communication. We extend a previously established two-way comparison technique to obtain all data at the local site. Two optical carrier signals are injected into the bidirectional fiber from both ends, and one carrier is reflected back from the remote end. This enables the phase comparison of the two carrier signals at a single site without the need to transmit experimental data. The common-mode frequency noise induced by the bidirectional fiber link is detected and effectively suppressed without the need for sophisticated active fiber noise control. Our demonstration system, which uses a 260 km field fiber link and a common laser source, achieves a fractional instability of $2.5\times 10^{-17}$ at 1 s averaging time and scales down to $3.5\times 10^{-21}$ at 8000 s. This scheme offers the distinct advantage of completing the comparison at a single site, eliminating the need for remote data transfer via communication. This method is expected to enhance reliability for high-precision frequency comparisons between remote optical clocks and advanced atomic clocks.

## 1. Introduction

In recent decades, transferring and comparing the high-precision time or frequency references between atomic clocks at different places has become increasingly important for many research areas, such as physical metrology [1], fundamental physics [2,3], timekeeping [4], and relativistic geodesy [5]. Current optical clocks have reached an uncertainty and instability to the $10^{-18}$ level [6,7], and the new type of nuclear clocks could be even better [8,9]. When comparing the performance between different clocks, the uncertainty for the carrier-phase two-way satellite time and frequency transfer (TWSTFT) method is about $10^{-13}$ at 1 s and $10^{-16}$ for 1 day integration time [10]. In 2013, a 920 km fiber link between Physikalisch-Technische Bundesanstalt (PTB) and the Max–Planck Institute of Quantum Optics (MPQ) was demonstrated. The work paved the way for long-distance optical frequency transfer at a 1000 km scale [11]. In 2015, a 1480 km optical fiber link between France and Germany was implemented for the first remote optical clock comparison, and this work made it possible to compare future optical clocks at the $10^{-19}$ level after only a few hours [12]. In addition, there have been many gratifying developments in the study of optical frequency [13,14,15,16,17].

Focusing on high-precision remote frequency reference comparison, Calosso et al. proposed a two-way optical phase comparison approach instead of the conventional frequency transfer technique [18]. In their system, the two optical carrier signals to be measured are injected into a bidirectional fiber from both ends. The transferred carriers are compared with the local references at the remote sites. The common fiber-induced noise can be rejected by combining both phase comparisons at both sites. Theoretically, a 3 dB improvement in uncertainty can be achieved [19]. At the same time, active fiber noise compensation is abandoned, which makes the comparison system simpler and more robust. In Ref. [19] and the system proposed by Bercy et al. in [20], two frequency measurement systems were implemented in a two-way comparison of the two ends of a fiber link when they were located in the same lab for demonstration purposes. To complete the measurement of two distant clocks, the relative frequency information should be transferred by an extra data link to the remote user. A hybrid approach was demonstrated in 2017 to look at the main constraints on the two-way comparison in a 43 km fiber link [21]. The team provided a data set of unidirectional optical frequency transfer over an urban fiber link of 2×43 km with a half-year’s worth of data in 2020. The data set has a relative frequency instability of $8.0\times 10^{-16}$ at 1 s integration time [22]. A potential issue may occur when the two-frequency measurement system needs to be synchronized to make a simultaneous measure of the optical frequencies at both sites. In our previous work, to simplify the remote bidirectional comparison scheme, we proposed a method called local two-way phase comparison in a 50 km and 200 km fiber spool to verify the feasibility of the experiment [23,24]. However, the comparison between remote optical clocks must be carried out in field optical fibers, which may face more complex noise situations.

In this paper, we detail a system for optical frequency comparison over a 260 km field optical fiber link, building upon prior research. The frequency comparison system was set up in a laboratory setting, utilizing just one photodetector (PD) and a handful of radio frequency devices at the local site to gather necessary data. We compared the phases of the two carrier signals at a single site to eliminate the fiber noise. In general, the single-carrier bidirectional propagation technique requires a complex active noise cancellation (ANC) system at the local site to detect and eliminate fiber phase noise, such as a PID feedback system or active temperature control system. However, our system can passively eliminate fiber link noise and common mode noise caused by lasers at the local site and has a simpler structure and stronger robustness. This approach enables data collection for measurement without relying on the synchronization of instruments that are geographically separated, ultimately improving comparison precision. By comparing the optical frequency, the relative instability of the field fiber link is $2.5\times 10^{-17}$ at 1 s and $3.5\times 10^{-21}$ at 8000 s integration time. The results demonstrate the significant potential of comparing optical reference signals located in distant locations and will be crucial for conducting precise and high-resolution optical frequency comparisons between two remote optical clocks using straightforward electronic devices.

## 2. Experimental Principle

**Figure 1:**
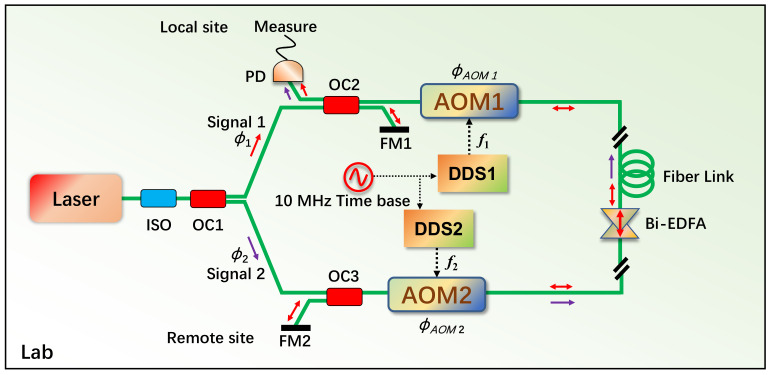
Schematic diagram of fiber noise detection and comparison. ISO: fiber isolator, OC: optical coupler, PD, photodetector, FM: Faraday mirror, AOM: acousto-optical modulator, Bi-EDFA: bidirectional erbium-doped fiber amplifier, DDS: direct digital synthesizer.

In this study, we tested the effectiveness of a local two-way fiber noise detection and comparison method using a 260 km field fiber to assess its overall stability. The experimental setup can be seen in Figure 1. During the experiment, the common-mode phase noise of the bidirectional fiber links was effectively eliminated through the simultaneous transfer of two-way optical signals, without needing active compensation for the fiber noise. By synchronously measuring the two beat-notes against a local laser at a single site, we were able to cancel out any potential noise without the need for remote data communication.

To fully investigate the potential of the local two-way scheme, we employed a custom-built ultrastable 1.55 µm laser with a line width of 1.9 Hz, and the frequency stability is at about the $10^{-15}$ level as the common laser source for both directions [25]. The laser output was split into two parts, designated as signal 1 and signal 2, after passing through an isolator (indicated in Figure 1 with red and purple arrows, respectively). This configuration allows us to neglect the contribution of frequency noise from the laser sources. The laser outputs were coupled into the fiber from the local site, transferring light to the remote site. At the local site, part of the light was reflected into the photodiode (PD) by the first Faraday mirror (FM 1), while the remaining light was coupled into the first acousto-optic modulator (AOM 1), which was driven at a frequency of 110 MHz ($f_{1}$) to shift the optical frequency and filter out stray reflections from connectors and splices. The signal light was then reflected by the second Faraday mirror (FM 2) and returned to the local site, where the beat-note A between signal 1 and itself after a round trip was detected by the PD at the beat-note B frequency, 2($f_{1}$ + $f_{2}$). Simultaneously, the light from signal 2 at the remote site was coupled into the fiber link after the second AOM 2, which was driven at a frequency of 50 MHz ($f_{2}$). Upon reaching the local site, the beat-note B between signals 1 and 2 was detected by the PD at the beat-note A frequency ($f_{1}$ + $f_{2}$). By carefully selecting the AOM driving frequencies, we could easily filter and separate the beat-note signals A and B. Furthermore, in our experimental setup, the drive frequencies for the AOMs were generated by two direct digital synthesizers (DDSs) with a reference signal from the same 10 MHz time base. This system required only a few components: two FMs, two AOMs, and one PD, making its structure simple and cost-effective.

Based on the scheme presented above, the phase noises of two beat-note signals $\phi_{A}$ and $\phi_{B}$ can be written as follows:

(1) $$\begin{array}{cc} \phi_{A}=\; & \phi_{1}+\phi_{AOM1}+\phi_{1\to 2}\left(t-\tau \right)+\phi_{AOM2}+\phi_{2\to 1}\\ \; & +\phi_{AOM2}+\phi_{AOM1}-\phi_{1}\\ =\; & 2\left(\phi_{AOM1}+\phi_{AOM2}\right)+\phi_{1\to 2}\left(t-\tau \right)+\phi_{2\to 1}, \end{array}$$

and

(2) $$\begin{array}{cc} \phi_{B}=\; & \phi_{2}+\phi_{AOM2}+\phi_{2\to 1}+\phi_{AOM1}-\phi_{1}\\ =\; & \left(\phi_{2}-\phi_{1}\right)+\phi_{AOM1}+\phi_{AOM2}+\phi_{2\to 1}. \end{array}$$

In Equations (1) and (2), where τ is the light signal single-trip delay time in the fiber link, referred to as $\tau =L/c_{n}$, $c_{n}$ is the effective speed of light in the fiber link, and *L* is the length of the field fiber link. $\phi_{1}$ and $\phi_{2}$ are the phase noises of signals 1 and 2, respectively, $\phi_{AOM1}$ and $\phi_{AOM2}$ are the phase noises of AOM 1 and AOM 2, respectively, and $\phi_{1\to 2}$ and $\phi_{2\to 1}$ are the fiber phase noises caused by the bidirectional fiber link. Combining Equations (1) and (2) in postprocessing, we have

(3) $$\phi_{B}-\frac{1}{2}\phi_{A}=\left(\phi_{2}-\phi_{1}\right)+\frac{1}{2}\phi_{2\to 1}-\frac{1}{2}\phi_{1\to 2}\left(t-\tau \right).$$

Equation (3) indicates that when the phase noises of AOM 1 and AOM 2 are disregarded, the outcome of the phase comparison is composed of the laser’s phase difference $\left(\phi_{2}-\phi_{1}\right)$ and the fiber’s phase noise. Under ideal conditions, where signal 1 and signal 2 both stem from the same laser source, it is reasonable to assume that $\phi_{2}-\phi_{1}=0$. When $\left(\phi_{2}-\phi_{1}\right)$ is eliminated in Equation (3), the phase comparison result becomes exclusively sensitive to the fiber’s phase noise [20]. We know that

(4) $$\phi_{1\to 2}\left(z,t\right)=\int_{0}^{L}\delta \phi \left[z,t-\left(\tau -\frac{z}{c_{n}}\right)\right]dz,$$

(5) $$\phi_{2\to 1}\left(z,t\right)=\int_{0}^{L}\delta \phi \left[z,t-\frac{z}{c_{n}}\right]dz,$$

where δϕ is the phase perturbation per unit of length at coordinate *z* at time *t*. Hence, we express the two-way comparison phase noise $\phi_{two}\left(z,t\right)$ as

(6) $$\phi_{two}\left(z,t\right)=\phi_{B}-\frac{1}{2}\phi_{A}\approx \frac{1}{2}\left[\phi_{2\to 1}-\phi_{1\to 2}\left(t-\tau \right)\right].$$

We transform Equation (6) into the frequency domain using a Fourier transform as

(7) $${\tilde{\phi }}_{two}\left(\omega \right)=\int_{0}^{L}e^{-j\omega \tau }\left[j\sin\omega \left(\tau -\frac{z}{c_{n}}\right)\right]\delta \tilde{\phi }\left(z,\omega \right)dz,$$

The phase noise power spectral density (PSD) $S_{two}\left(\omega \right)$ of this signal is [23]

(8) $$\begin{array}{cc} S_{two}\left(\omega \right)= & \frac{S_{fiber}\left(\omega \right)}{L}\int_{0}^{L}\frac{1}{2}\left[1-\cos2\omega \left(\tau -\frac{z}{c_{n}}\right)\right]dz\\ = & \frac{1}{2}S_{fiber}\left(\omega \right)\left[1-\sec\left(2\omega \tau \right)\right]\\ = & \frac{1}{3}S_{fiber}\left(\omega \right)\omega^{2}\tau^{2}. \end{array}$$

Due to sec(0)≈1, the suppression of bidirectional phase noise is more effective at lower frequencies, with its level being approximately half of the one-way phase noise at higher frequencies. It is presupposed that the noise power spectral density (PSD) is independent of *z* [26], and thus, Equation (8) is valid within the spectral domain where 4fπτ≪1. Notably, Equation (8) resembles the formula derived for an optical link with active noise compensation [27]. This similarity implies that a two-way optical phase comparison link offers greater resilience without necessitating the implementation of a complex active fiber noise.

## 3. The Experimental Setup

### 3.1. The 260 km Field Fiber Link

**Figure 2:**
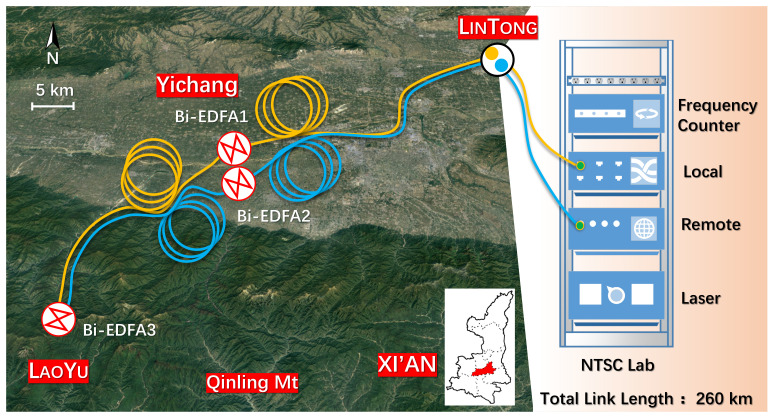
The routing map of 260 km field fiber link (based on Baidu map).

We conducted the test on the communication operator’s 260 km field optical fiber link in order to further evaluate the functionality of the two-way phase comparison system and bring the testing environment closer to the project’s actual application scenario. The routing diagram of the field link is shown in Figure 2 (based on the Baidu map). The optical fiber starts from the LinTong Laboratory of the National Time Service Center (NTSC) and passes through the Yichang Telecommunications room in Xi’an City to the LaoYu Telecommunications room in the Qinling Mountains. The one-way length of the link is about 130 km. The optical fiber returns to the LinTong laboratory along the second optical fiber of the same cable after looping back in the LaoYu Telecommunications room, so the total length of the link is 260 km (marked on Figure 2 with yellow and blue lines). With a distance of approximately 60 km, the optical fiber is partially buried and partially put on the pole as it travels from LinTong Laboratory to Yichang Telecommunications in Xi’an. About 70 km of buried optical cable connects the Yichang Telecommunications room in Xi’an with the LaoYu Telecommunications room. The total loss of the field fiber link is about 60 dB. In order to compensate for the fiber link loss as much as possible, two Bi-EDFAs with a gain of 16 dB are arranged in the Yichang Telecommunications room in Xi’an, and one Bi-EDFA with a gain of 20 dB is arranged in the LaoYu Telecommunications room. In addition, since the majority of the optical fibers between LinTong and Laoyu are installed along highways or other major thoroughfares, their environment is constantly changing, their phase noise is complex, and they occasionally experience interference like cycle slip. In this experiment, the laser source, optical system (including the local site and remote site, see Figure 1), and measuring device are all placed in the same cabinet in the NTSC laboratory.

### 3.2. The Data Post-Processing

**Figure 3:**
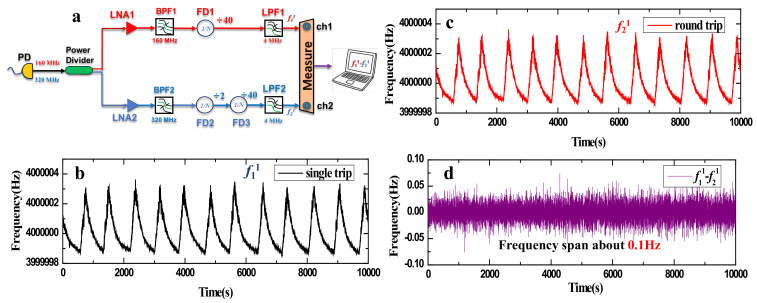
(**a**) The schematic diagram of data processing; PD: photodetector, LNA: low-noise amplifier, BPF: band-pass filter, FD: frequency divider, LPF: low-pass filter, ch: frequency counter channel; (**b**) the single-trip frequency curve; (**c**) the round-trip frequency curve; (**d**) the data postprocessing curve.

The signal processing process is shown in Figure 3a. The 160 MHz signal (beat-note A) in the experiment is the beat-note frequency (beat-note B) after the single-trip transmission of the signal light, while the 320 MHz signal is the beat-note frequency after round-trip transmission. Due to the small signal amplitude, the detector’s signal must first be split into two equal parts using a power divider. The two parts of the signal are then amplified using low-noise amplifiers (LNAs), and the two frequency signals are separately extracted using spiral band-pass filters (BPFs) with center frequencies of 160 MHz and 320 MHz, respectively. The 160 MHz signal is divided into $f_{1}^{1}$ = 4 MHz with a 40 times frequency divider (FD1), while the 320 MHz signal is divided into 160 MHz with a 2 times frequency divider (FD2) and then into $f_{2}^{1}$ = 4 MHz with a 40 times frequency divider (FD3). Before being used for measurement, two channels of 4 MHz signals must pass through with a 5 MHz low-pass filter (LPF) that eliminates the harmonic produced by frequency division. The reason for dividing the measured signal to 4 MHz is that the measurement bandwidth of the frequency counter (FXE K+K) is about 65 MHz, and the measured low frequency data need to be converted to the frequency before the frequency division during processing, otherwise there will be errors in calculating the noise.

The part frequency data measured by frequency counter are also shown in Figure 3. The black curve in Figure 3b is the single-trip beat frequency information from the measured remote site to the local site, and the red curve in Figure 3c is the round-trip beat frequency information from the measured local site to the remote site and then returned through the field fiber link. It can be seen that the central value of the two groups of measured frequency information is 4 MHz. In Figure 3b,c, there is obvious frequency modulation information, and the modulation period is about 800 s, resulting in the form of a saw-tooth wave. This is mostly because the phase comparison system is free-running and lacks either active or passive temperature control, causing the temperature of the lab air conditioner to have an impact on the frequency information. The cooling time of the air conditioner is shorter than the natural heating process, so the saw-tooth wave has different slopes before and after the peak. Then, we compare and process the collected frequency data, that is, $f_{1}^{1}-f_{2}^{1}$ is shown by the purple curve in Figure 3d. From the results, it can be seen that because the two groups of data used for comparison have the same temperature modulation information, the common mode modulation is completely removed after data processing, and only the residual noise in the link is left. At this time, the width range of frequency difference is only about 0.1 Hz.

## 4. Results and Discussion

**Figure 4:**
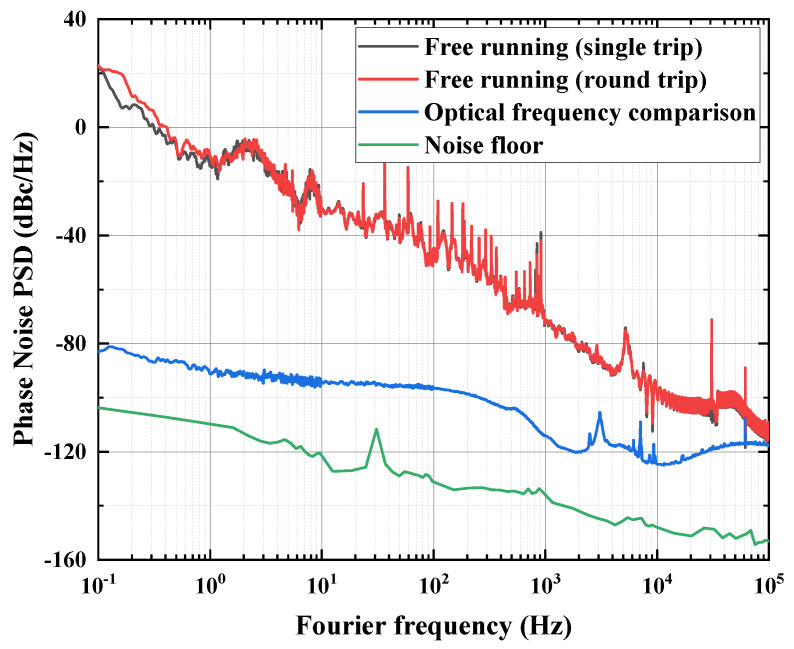
The phase noise of 260 km field fiber link; the black curve is the free running of a single trip, the red curve is the free running of a round trip, the blue curve is the optical frequency comparison, and the green curve is the system floor.

With the utilization of single bidirectional fiber and synchronous phase measurement, beat-note A exhibits double-fiber phase noise due to the round-trip travel of light in the fiber link. Consequently, it can be halved and juxtaposed with beat-note B. During data postprocessing, the phase noise can be effortlessly nullified. The power spectral density (PSD) of phase noise in our system is depicted in Figure 4. Due to restrictions of the measuring equipment, the phase noise PSDs of one-way fiber noise for round-trip and single-trip signals (black curve and red curve, respectively) are not simultaneously taken, resulting in noncoincident curves. Among them, the spikes between 1 Hz and 10 Hz are low-frequency phase noise caused by human daily activities (such as vibration) coupled with fiber-optical links. The dense spikes between 10 Hz–1 kHz are caused by high-frequency phase noise due to traffic factors (such as car honking, etc.) or other sporadic interference. During the evaluation of our system’s performance, these phase noise PSDs coincide from 0.1 Hz to 100 kHz, thereby facilitating easy rejection of common phase noise in the two beat-note signals using the two-way configuration. Therefore, the phase noise PSD of the two-way phase comparison (blue curve) displays a smooth amplitude around −90 dBc/Hz within Fourier frequencies of 1 Hz to 100 Hz. This value is nearly four orders of magnitude lower than one-way fiber noise at 1 Hz and is primarily white noise. Additionally, the noise floor of the comparison system is indicated by the green curve, showcasing smaller values compared to the comparison outcomes as demonstrated in Figure 4.

Our findings support that the optical phase comparison system we developed aligns with the theoretical projections and effectively eliminates phase noise. The experimental setup is immune to interference from the interferometer’s transfer function, enabling wide bandwidth measurement of phase noise, particularly for broadband signals. It is crucial to note, however, that the conclusions drawn from Equation (8) are based on gradual variations in light intensity. In real-world scenarios, there is constant, rapid fluctuation in light levels that our system cannot entirely eradicate, thereby reducing the accuracy of our comparisons. Therefore, a certain degree of measurement bandwidth remains inherent in our system.

**Figure 5:**
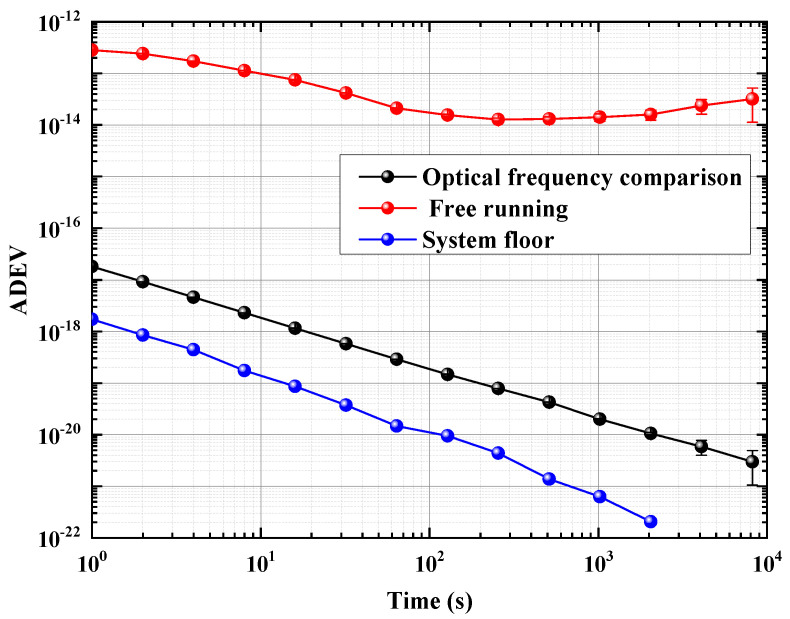
The ADEV of 260 km field fiber link; the red-dot line is the free running, the black-dot line is the optical frequency comparison, and the blue-dot line is the system floor.

In the optical frequency comparison system, the factors affecting the transfer instability mainly include the phase noise caused by vibration or temperature variation. The impact of vibration mainly leads to the short-term cycle slip of phase data, leading to the overall deterioration of instability results, while the temperature drift mainly leads to the long-term drift of phase data, leading to the deterioration of long-term instability. In our 260 km field optical phase comparison transfer link, the phase noise caused by temperature fluctuation and vibration was eliminated through data postprocessing. In Figure 5, the Allan deviation (ADEV) illustrates the relative frequency instability of the optical phase comparison within a 260 km fiber link. The instability is depicted for both one-way fiber noise (represented by the red dotted line) and two-way phase comparison (represented by the black dotted line). The stability of the two-way bidirectional comparison can achieve an impressive low of $2.5\times 10^{-17}$ at 1 s on average, and it even reaches $3.5\times 10^{-21}$ at an integration time of 8000 s with a 1/τ slope. The system floor is indicated by the blue dotted line.

## 5. Conclusions

In summary, we introduced a novel local method for detecting and comparing optical frequency noise in a 260 km field optical fiber without the need for synchronous remote measurement of optical phase. This method effectively eliminates fiber noise by utilizing bidirectional propagation within a single fiber and is not affected by phase noise in the fiber link. The resulting relative frequency instability is $2.5\times 10^{-17}$ at 1 s and $3.5\times 10^{-21}$ at 8000 s integration time. The optical frequency noise detection and comparison system is robust and does not require a complex active fiber noise control loop. Additionally, this approach allows for on-site completion of comparisons without the need for remote data transfer via communication, making it possible to compare optical signals using simple electronic devices. This bidirectional setup offers a more reliable method for high-precision frequency comparison between remote optical clocks and advanced atomic clocks compared to traditional techniques.

## Data Availability

The data presented in this study are available on request from the corresponding author.
